# Health-related quality of life in long-term survivors of unresectable locally advanced non-small cell lung cancer

**DOI:** 10.1186/s13014-017-0909-6

**Published:** 2017-12-02

**Authors:** Juntao Ran, Jingbo Wang, Nan Bi, Wei Jiang, Zongmei Zhou, Zhouguang Hui, Jun Liang, Qinfu Feng, Luhua Wang

**Affiliations:** Department of Radiation Oncology, National Cancer Center/Cancer Hospital, Chinese Academy of Medical Sciences and Peking Union Medical College, 17 Panjiayuan Nanli, Chaoyang District, Beijing, 100021 People’s Republic of China

**Keywords:** Health-related quality of life, Non-small cell lung cancer, Survivor, Radiotherapy, Chemotherapy

## Abstract

**Background:**

Heath-related quality of life (HRQoL) among survivors with unresectable locally-advanced non-small cell lung cancer (LA-NSCLC) treated with radiotherapy and chemotherapy still is not clear. The current study were performed to determine HRQoL for long-term survivors with unresectable LA-NSCLC and to identify risk factors for poor HRQoL.

**Methods:**

Among patients with LA-NSCLC receiving radiotherapy and chemotherapy between January 2006 and December 2010, 82 long-term survivors beyond 5 years were identified in this cross-sectional study. The European Organization for Research and Treatment of Cancer Quality of Life Questionnaire (EORTC QLQ)-C30 and the lung cancer-specific questionnaire QLQ-LC13 were employed to gather information on HRQoL. HRQoL scores were compared between different subgroups to analyze factors related to HRQoL.

**Results:**

Fifty-five out of 82 (67%) long-term survivors completed the HRQoL survey. They reported a mild reduction in global health status and physical and emotional functioning. Fatigue, dyspnea, coughing, and financial difficulties ranked the highest scores in the symptom scales. Analysis of risk factors for HRQoL showed age, exercise, smoking status, and treatment regimen were associated with global health status and functional scores, while age, gender, radiation pneumonitis, weight loss, and exercise were associated with symptom scores.

**Conclusions:**

This study provides the first description of the HRQoL of long-term LA-NSCLC survivors receiving radiotherapy and chemotherapy who may experience a relatively high HRQoL. Factors related to poorer HRQoL are potential targets for intervention.

## Background

For decades, health practitioners have improved the outcome of locally advanced NSCLC (LA-NSCLC) by using concurrent chemoradiotherapy. Thus, a substantial goal of LA-NSCLC management is to maintain or improve the survivors’ health-related quality of life (HRQoL). Information about the HRQoL of long-term LA-NSCLC survivors is essential to anticipate consequences and problems. Moreover, it is useful to analyze factors that affect HRQoL and find optimal intervention to improve HRQoL.

Long-term lung cancer survivors experienced quality of life (QoL) impairment in 35% of cases and reported declined QoL and a worsened symptom load [1]. A cross-sectional study of QoL showed that a distressed mood was the most important predictor of the QoL of long-term NSCLC survivors [2]. Most patients in both studies were stageIand treated with surgery. However, little is known about the HRQoL status of long-term LA-NSCLC survivors who have survived beyond 5 years receiving radiotherapy and chemotherapy. This study aimed to determine the HRQoL of long-term LA-NSCLC survivors receiving radiotherapy and chemotherapy and to explore the relationships between demographic, clinical characteristics, and HRQoL.

## Methods

### Patients

Patients diagnosed with histologically/cytologically confirmed LA-NSCLC (stages IIIA or IIIB) and treated with radiotherapy in our department between January 2006 and December 2010 were reviewed in the current study. We defined patients who are still living at least five years after diagnosis with LA-NSCLC as long-term survivors. Patients’ demographics and clinical characteristics were uniformly collected from their medical records. Tumor stages of all patients were reclassified using the 7th edition of the American Joint Committee on Cancer Staging. Eligibility included histologically or cytologically confirmed NSCLC, older than age 18 at the time of diagnosis, and medical records indicating at least 60 months of follow-up. No specific post-treatment rehabilitation program was provided for all eligible patients, but the smokers were encouraged to stop smoking. The protocol of this study was approved by the ethics committees of the institution.

### Assessment of HRQoL

Health-related QoL was assessed using a validated, cancer-specific core questionnaire, the European Organization for Research and Treatment of Cancer (EORTC) QLQ-C30 (version 3.0) and a lung-specific module, the EORTC QLQ LC-13 [3]. Questions were scaled and scored using the recommended EORTC quality of life group procedures [4]. All scores on the EORTC QLQ-C30 and the QLQ-LC 13 were transformed to a 0 to 100 scale according to the guidelines of EORTC. Higher scores for the global QL and functional domains indicate better global health and function, while higher scores for symptoms indicate greater symptom severity. Given that a 10-point change in each of the EORTC multi-item scales is generally considered to be a moderate or meaningful change [5, 6], for the single-item scales, four levels of responses were defined: not at all, a little, quite a bit, and very much. Demographic characteristics were collected at the instructions of the survey. Smoking status was classified as current, former, and never smoker. Pack years of smoking were calculated as the number of packs of cigarettes smoked per day during the years of smoking. Exercise was defined as at least 30 min of moderate aerobic exercise a day, at least five days a week.

### Survey procedures

Eligible patients were asked to sign the consent form if they would be interested in the survey and complete Chinese versions of the questionnaires, if feasible, during their latest visit to the hospital. Others were mailed a consent form, questionnaires, and a stamped envelope. After the letter, a trained interviewer telephoned the patient to ask if the survivor was interested in the survey. Those who agreed to take part in the survey either completed a letter in reply or responded through a trained interviewer via telephone for survivors who could not complete the questionnaires by themselves.

### Statistical analysis

The chi-squared test was applied for categorical variables to evaluate whether survey respondents differed significantly from nonrespondents of all patients. Cumulative survival was estimated according to the Kaplan-Meier method. Descriptive statistics, as appropriate, were used to provide a profile of HRQoL. HRQoL data were analyzed using nonparametric methods because the data were not normally distributed. Univariate data were compared by applying the Mann-Whitney *U* test or the KrusKal-Wallis test. Statistical analyses were performed using statistical software SPSS 19.0. The level of statistical significance was set to 0.05.

## Results

### Patient characteristics

The records of 505 patients with LA-NSCLC who underwent radiotherapy and chemotherapy were retrospectively reviewed. Of the 82 patients who were still living at 5 years or more after diagnosis, 55 patients completed the Chinese versions of the EORTC QLQ-C30 questionnaires and the QLQ-LC13 module. Patients’ characteristics are summarized in Table 1. Those who completed the survey (Survey Completed Group, SCG) included more patients treated with concurrent chemoradiotherapy (CCRT) and higher objective response rates than those who did not complete the survey (non-Survey Completed Group, nSCG) (*p* < 0.05), but they did not differ with regard to other factors.

**Table 1:** Comparison of characteristics of patients between nSCG and SCG

Characteristics		nSCG (450) n (%)	SCG (55) n (%)	*P* value
Age	Mean	61	60	*0.563*
	SD	11	10	
Gender	Male	372 (82.7%)	45 (81.8%)	*0.876*
	Female	78 (17.3%)	10 (18.2%)	
KPS Score^a^	90–100	116 (25.8%)	19 (34.5%)	*0.165*
	60–80	334 (74.2%)	36 (65.5%)	
Histology	Squamous	266 (59.1%)	28 (50.9%)	*0.244*
	Non-squamous	184 (40.9%)	27 (49.1%)	
Stages (AJCC 2002)	IIIA	156 (34.7%)	25 (45.5%)	*0.115*
	IIIB	294 (65.3%)	30 (54.5%)	
Smoking status	Never	89 (19.8%)	13 (23.6%)	*0.501*
	Former/current	361 (80.2%)	42 (76.4%)	
Weight loss^b^	≥5 %	121 (26.9%)	15 (27.3%)	*0.959*
	<5 %	328 (73.1%)	40 (72.7%)	
Regimens	RT	122 (27.1%)	5 (9.1%)	*0.006*
	CRT	147 (32.7%)	18 (32.7%)	
	CCRT^c^	181 (40.2%)	32 (58.2%)	
RT dose	<60Gy	138 (30.7%)	11 (20%)	*0.102*
	≥60Gy	312 (69.3%)	44 (80%)	
RT-technique	2D–RT	41 (9.1%)	2 (3.6%)	*0.343*
	3D–CRT	22 (4.9%)	2 (3.6%)	
	IMRT	387 (86%)	51 (92.7%)	
Response rate^d^	CR + PR	285 (64.2%)	43 (78.2%)	*0.039*
	SD + PD	159 (35.8%)	12 (21.8%)	
Radiation esophagitis^e^	<grade 2	269 (64.4%)	37 (67.3%)	*0.670*
	≥grade 2	149 (35.6%)	18 (32.7%)	
Radiation pneumonitis^f^	<grade 2	330 (85.3%)	46 (83.6%)	*0.750*
	≥grade 2	57 (14.7%)	9 (16.4%)	

^a^one patient <80 in SCG, ^b^one patient without data of weight loss, ^c^CCRT including:induction chemotherapy + CCRT,CCRT + consolidation chemotherapy,induction + CCRT + consolidation chemotherapy and CCRT, ^d^six patients without data of response rate, ^e^32 patients without data of radiation esophagitis, ^f^63 patients without data of radiation pneumonitis. *CCRT* Concurrent chemo-radiotherapy, *nSCG* non-Survey completed group, *RT* Radiotherapy, *SCG* Survey completed group

The demographic characteristics at the time of the interview of the 55 patients in SCG group are listed in Table 2. Thirty-one patients reported no comorbidities. Three patients had second primary cancer (lung cancer, rectal cancer, and bladder cancer, respectively), two patients had metastasis (brain and liver, respectively), and five patients received target therapy (3 with erlotinib and 2 with gefitinib). The majority (54/55, 98%) could perform their daily life activities by themselves (KPS ≥ 80). They also reported their educational level, employment status, income per month, and exercise status. Main types of aerobic exercise reported were brisk walking, jogging, Tai chi, and Qigong. All 55 patients had health insurance.

**Table 2:** Demographic characteristics of 55 long-term survivors

Characteristic	n (%)
Comorbidities	
Emphysema/COPD	4 (7.3%)
Heart disease	7 (12.7%)
Hypertension	11 (20%)
Diabetes	4 (7.3%)
Cancer	
Second primary cancer	3 (5.5%)
Metastasis	2 (3.6%)
Other comorbidities	5 (9.1%)
Target therapy	5 (9.1%)
Smoking status	
Never	13 (23.6%)
Former	38 (69.1%)
Current	4 (7.3%)
< 20 pack-y	21 (38.2%)
≥ 20 pack-y	34 (61.8%)
Education	
Less than high school	21 (38.2%)
High school graduate or more	34 (61.8%)
Employment status	
Employed	10 (18.2%)
Retired and unemployed	45 (81.8%)
Income per month	
< 3000	16 (29.1%)
≥ 3000	39 (70.9%)
Medical insurance	
URBMI	51 (92.7%)
NRCMS	4 (7.3%)
Taking exercise	
yes	45 (81.8%)
no	10 (18.2%)

*URBMI* Urban resident basic medical insurance, *NRCMS* New rural cooperative medical scheme

### Survival and compliance

Of 505 patients, the median survival time was 21 months (95% CI, 19.14 to 22.86 months), and the overall 1, 2, and 5-year survival rates were 73.2%, 44.4%, and 17.1%, respectively. Because of death or no follow-up, only 55 of the 82 (67%) long-term survivors completed the survey. Fourteen patients died, including 2 patients who died from pancreatitis and a heart attack, respectively in the 5th year. Besides those patients, there were 9 patients lost to follow-up and 4 patients who did not want to complete the questionnaires.

### Health-related quality of life

**Fig. 1:**
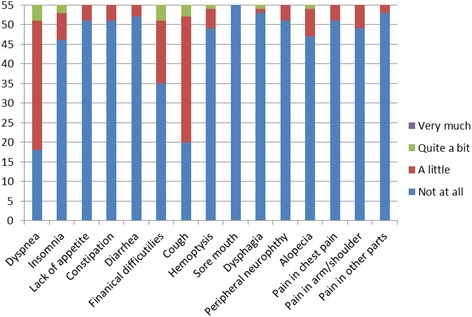
Number of patients in four levels of responses of single-item symptom scales

Self-reported HRQoL scores for the QLQ-C30 and the QLQ-LC13 are displayed in Table 3. The greatest impairment was seen in global health status (76.67 ± 15.91), followed by physical impairment (86.06 ± 14.53), and emotional (89.58 ± 14.65) functioning. There were relatively high levels of role, social, and cognitive functioning with scores above 90. Most symptom scores were very low, except for scores of fatigue (27.02 ± 15.72), dyspnea (24.85 ± 19.48 in QLQ-C30; 18.08 ± 13.74 in QLQ-LC13), coughing (23.03 ± 19.11), and financial difficulties (14.55 ± 21.05). The patients of four levels of responses of single-item symptom scales are shown in Fig. 1. The most prevalent symptoms were fatigue (49/55, 89%), dyspnea (QLQ-LC13, 48/55, 87.3%; QLQ-C30, 37/55, 67.3%), coughing (35/55, 63.6%), and financial difficulties (20/55, 36.4%).

**Table 3:** HRQoL Scores of Long-term LA-NSCLC Survivors

QoL Scale	Mean (SD)	Median (IQR)	Range
QLQ-C30			
Global health status/QoL	76.67 (15.91)	83.33 (66.67–91.67)	(25–100)
Physical functioning	86.06 (14.53)	93.33 (80.0–93.3)	(20–100)
Role functioning	91.21(17.53)	100 (83.3–100)	(0–100)
Emotional functioning	89.58 (14.65)	100 (75.0–100)	(50–100)
Cognitive functioning	94.24 (12.51)	100 (100–100)	(33.3–100)
Social functioning	93.94 (13.75)	100 (100–100)	(33.3–100)
Fatigue	27.02 (15.77)	33.33 (22.22–33.33)	(0–100)
Nausea and vomiting	0.40 (2.99)	0 (0–0)	(0–22.22)
Pain	5.76 (12.92)	0 (0–0)	(0–66.67)
Dyspnea	24.85 (19.48)	33.33 (0–33.33)	(0–66.67)
Insomnia	6.67 (16.23)	0 (0–0)	(0–66.67)
Appetite loss	2.42 (8.74)	0 (0–0)	(0–33.33)
Constipation	2.42 (8.74)	0 (0–0)	(0–33.33)
Diarrhea	1.82 (7.64)	0 (0–0)	(0–33.33)
Financial difficulties	14.55 (21.05)	0 (0–33.33)	(0–66.67)
QLQ LC-13			
Dyspnea	18.08 (13.74)	11.11 (11.11–22.22)	(0–66.67)
Coughing	23.03 (19.11)	33.33 (0–33.33)	(0–66.67)
Hemoptysis	4.24 (12.92)	0 (0–0)	(0–66.67)
Sore mouth	0 (0)	0 (0–0)	(0–0)
Dysphagia	1.82 (9.98)	0 (0–0)	(0–66.67)
Peripheral neuropathy	2.42 (8.74)	0 (0–0)	(0–33.33)
Alopecia	5.45 (14.00)	0 (0–0)	(0–66.67)
Pain in chest	2.42 (8.74)	0 (0–0)	(0–33.33)
Pain in arm/shoulder	3.64 (10.49)	0 (0–0)	(0–33.33)
Pain in other parts	1.21 (6.30)	0 (0–0)	(0–33.33)

*IQR* Interquartile range, *SD* Standard deviation

### Risk factors for poor HRQoL

In global health status and functional scales, survivors of a younger age (≤60 years old), exercise, without comorbidity, and never smoker were significantly associated with more favorable scores for global health status. Likely, those with age (≤60 years old), exercise and without comorbidity had better physical, role and social functioning (*p* < 0.05). In contrast, CCRT was negatively associated with emotional functioning (*p* < 0.05) (Table 4). On the examination of the association between symptom load and the characteristics of survivors, fatigue was significantly negatively affected by weight loss <5% and lack of exercise (*p* < 0.05), while dyspnea was significantly negatively affected by gender: male, with at least one comorbidity, radiation pneumonitis (RP, ≥ grade 2), and age (>60 years old) (*p* < 0.05) (Table 5). HRQoL was not significantly affected by other factors.

**Table 4:** Relationship between selected demographic/clinical features and global health status, functional scale scores, mean (SD)

	Age		*p*	Exercise		*p*	Comorbidity		*p*	Smoking status		*p*	Regimens			*p*
	≤60 (*n* = 25)	>60 (*n* = 30)		Yes (*n* = 45)	No (*n* = 10)		Yes (*n* = 24)	No (*n* = 31)		Never (*n* = 13)	Former/current (*n* = 42)		RT (*n* = 5)	RT + CT (*n* = 18)	CCRT (*n* = 32)	
Global health status	82.64 (12.98)	71.39 (16.48)	0.011	79.81 (14.81)	62.5 (13.18)	0.001	69.79 (17.34)	81.99 (12.56)	0.007	85.71(12.42)	73.58 (15.91)	0.01	73.33 (21.57)	79.63 (11.15)	75.52 (17.45)	0.775
Physical functioning	93.06 (7.22)	80.0 (16.33)	0.000	88.59 (13.9)	74.67 (12.09)	0.001	80.28 (17.42)	90.54 (10.00)	0.007	90.95 (8.52)	84.39 (15.82)	0.162	78.67 (17.26)	85.56 (11.72)	87.5 (15.59)	0.293
Role functioning	98.61 (4.71)	85.0 (21.60)	0.001	93.7 (17.51)	80.0 (13.15)	0.000	85.42 (22.69)	95.70 (10.00)	0.019	96.43 (9.65)	89.43 (19.28)	0.157	86.67 (18.26)	90.74 (13.06)	92.19 (19.85)	0.414
Emotional functioning	90.97 (13.66)	88.11 (15.63)	0.492	90.59 (14.53)	85.0 (15.11)	0.110	87.22 (16.90)	91.40 (12.64)	0.284	91.07 (14.05)	89.07 (14.99)	0.619	95.0 (11.18)	95.37 (9.58)	85.47 (16.31)	0.035
Social functioning	100 (0)	88.89 (17.14)	0.001	95.93 (12.39)	85.0 (16.57)	0.002	88.20 (18.04)	98.39 (6.60)	0.002	98.81 (4.45)	92.28 (15.41)	0.114	86.67 (21.73)	94.44 (9.90)	94.79 (14.32)	0.395

*CCRT* Concurrent chemoradiotherapy, *CT* Chemotherapy, *RT* Radiotherapy, *SD* Standard deviation

**Table 5:** Relationship between selected demographic/clinical features and symptom scale scores, mean (SD)

	Age		*p*	Gender		*p*	Comorbidity		*p*	Exercise		*p*	Weight loss (≥5%)		*p*	RP		*p*
	≤60 (n = 25)	>60 (n = 30)		Male (n = 45)	Female (n = 10)		Yes (n = 24)	No (n = 31)		Yes (*n* = 45)	No (*n* = 10)		Yes (*n* = 15)	No (*n* = 40)		<grade 2 (*n* = 46)	≥grade 2 (*n* = 9)	
Fatigue	23.15 (13.48)	30.28 (17.18)	0.110	26.85 (16.6)	27.78 (12.0)	0.491	29.98 (19.71)	24.73 (11.73)	0.190	25.68 (16.72)	33.06 (8.73)	0.026	17.04 (13.19)	30.76 (15.14)	0.001	25.3 (12.45)	35.8 (26.51)	0.255
LCDY	10.88 (9.62)	24.07 (14.02)	0.000	19.88 (13.74)	10.0 (11.05)	0.019	25.00 (15.46)	12.72 (9.43)	0.001	16.42 (12.64)	25.56 (16.6)	0.064	20.74 (9.26)	17.08 (15.06)	0.118	15.34 (9.74)	32.1 (21.83)	0.018

*LCDY* Dyspnea in QLQ LC13, *RP* Radiation pneumonitis, *SD* Standard deviation

## Discussion

Obtaining a satisfactory level of HRQoL is a fundamental goal of the therapy for LA-NSCLC patients. To better understand HRQoL in long-term LA-NSCLC survivors, based on QLQ-C30 and QLQ-LC13 using the most frequently used instruments in patients with lung cancer [7], we report the patient-reported HRQoL outcomes from the cross-sectional study for long-term LA-NSCLC survivors treated with radiotherapy and chemotherapy. Our findings suggest that long-term LA-NSCLC survivors have a relatively high HRQoL, while the long-term NSCLC survivors reported a slight reduction in global health status, physical and emotional functioning, and the predominant symptom load includes fatigue, dyspnea, coughing, and financial difficulties.

Our study shows that the 5-year overall survival rate of all patients was 17.1%; this figure is comparable with other studies [8, 9]. Survivors who completed the survey have a higher proportion of CCRT, and an objective response rate may result from a strong association between survival and treatment effect [10]. Several studies have documented the impact of cancer diagnosis and treatment on HRQoL in patients and short-term survivors with LA-NSCLC [11–13]. It is possible that the HRQoL of long-term LA-NSCLC survivors may differ from those experienced around diagnosis and treatment. Baseline global health status and functioning scores in two studies [11, 13] were lower than the current finding. Besides, this study found that long-term survivors of LA-NSCLC had a stable or slight decline in global health status and functioning scores; in contrast, for patients who just finished treatment or short-term survivors, global health status and all functioning scores except for emotional functioning declined significantly [11, 13]. The worsening of emotional functioning of long-term survivors is presumably an effect of a long-term burden on health, financial status, and their families. Furthermore, our study indicated that fatigue, dyspnea, coughing, and financial difficulties were the most frequent and high-score symptoms, while a high proportion of patients who had just finished treatment or short-term survivors experienced fatigue, loss of appetite, respiratory problems, cough, pain, and blood in sputum [14, 15]. These differences may relate to the symptoms associated with lung cancer, chemotherapy, and around the time of radiotherapy that were significant during treatment but later improved [11]. Financial problems are more common among the long-term survivors possibly due to long-term medical expenses though most of the patients have health insurance.

According to our assessment of the risk factors for a poor HRQoL in the present study, among long-term survivors, the HRQoL reported by younger survivors (≤60 years old) had significantly better scores in their global health status, physical, role and social functioning, and dyspnea than the older patients. The study [16] conducted by Lemonnier et al. also found that older age was associated with low HRQoL. In addition, our study indicated that exercise had a positive effect on global health status, physical, role and social functioning, and fatigue; the increasing number of evidence also suggests that exercise may be able to enhance cancer survivorship [17, 18]. Survivors treated with CCRT and with a smoking history had lower scores on emotional functioning and global health status, respectively, while patients with comorbidities had significantly more disruption in global health, functional scales, and dyspnea. These findings suggest that treatment regimen, tobacco use, and comorbidities have a long-term effect on HRQoL. As expected, survivors with RP (≥ grade 2) during or after radiotherapy had significantly higher dyspnea scores. Our results suggest that the risk factors of HRQoL include general (age), lifestyle (exercise), side effect (RP), and treatment (CCRT), which should be emphasized to further understand mechanisms and lead to tailored intervention strategies to improve HRQoL. In this study, smoking status was only associated with global health, but the symptom burden, particularly dyspnea and cough, continued partly due to a relatively small number of survivors who continued to smoke.

Several limitations should be considered when interpreting these results. First, our results have a potential bias in follow-up. Of the 82 patients, there were 4 patients (5%) who declined to answer the survey and 9 patients (11%) were lost to follow-up. It is generally accepted that those with advanced cancer do not complete surveys, leaving only those in better health to provide data. Hence, our results may overestimate HRQoL in these patients. Second, in this cross-sectional study, we did not observe longitudinal HRQoL, dynamic time-dependent changes. Thus, we could not define any change in HRQoL, and further studies are warranted. Finally, the number of patients was relatively small, thus limiting the statistical power of subgroup analyses and not correcting for multivariate comparisons.

## Conclusions

Long-term LA-NSCLC survivors treated with radiotherapy and chemotherapy have a relatively high HRQoL, with slight limitations in global health status and physical and emotional functioning and a higher symptom load of fatigue, dyspnea, coughing, and financial difficulties. Information about HRQoL of long-term LA-NSCLC survivors can be contributed to rehabilitation programs and long-term surveillance. Future prospective studies need to consider the long-term change of HRQoL and explore potential interventions.

## Abbreviations

CCRT: Concurrent chemoradiotherapy
CT: Chemotherapy
EORTC: European Organization for Research and Treatment of Cancer
HRQoL: Heath-related quality of life
IQR: Interquartile range
LA-NSCLC: Locally-advanced non-small cell lung cancer
LCDY: Dyspnea in QLQ LC13
NRCMS: New rural cooperative medical scheme’
nSCG: Non-Survey completed group
NSCLC: Non-small cell lung cancer
QLQ: Quality of life questionnaire
QoL: Quality of life
RP: Radiation pneumonitis
RT: Radiotherapy
SCG: Survey completed group
SD: Standard deviation
URBMI: Urban resident basic medical insurance
